# Liver abscess and endogenous endophthalmitis secondary to *Klebsiella variicola* in a patient with diabetes: first reported case

**DOI:** 10.5935/0004-2749.202200104

**Published:** 2022

**Authors:** Saúl Villoria Díaz, Jesús Alberto Piñuel González, Alicia Galindo-Ferreiro, Paola Stefanía Calles Monar, Ana María Alonso Tarancón, Marina Pilar González de Frutos

**Affiliations:** 1 Department of Ophthalmology, Complejo Asistencial Universitario de Palencia, Palencia, Spain; 2 Department of Ophthalmology, Rio Hortega University Hospital, Valladolid, Spain

Dear editor,

Endogenous endophthalmitis is a rare infection, representing 2%-16% of endophthalmitis cases. It arises from bacteremic seeding of the eye due to other infections such as endocarditis or liver abscess caused by common pathogens such as those in western countries including *Staphylococcus aureus* (25%), *Streptococcus* (30%-50%), and *Escherichia coli* (30%)^(1)^. Cases of endogenous endophthalmitis caused by *Klebsiella variicola* have not been previously reported.

We present the case of a 55-year-old female with type II insulin-dependent diabetes mellitus and with no relevant ophthalmological history. The patient presented to our emergency department with a sudden painful red eye and a decrease in vision in the right eye (RE) for 24 hours. She also complained of abdominal pain and a high fever. The recorded best-corrected visual acuity (BCVA) in the RE was hand motion (HM), and applanation tonometry revealed an intraocular pressure (IOP) of 60 mmHg. We found 4+ anterior chamber cells, exudative plaque covering the pupil (Figure 1A), and vitreous haze in the anterior segment examination. Although fundus was not visible due to pupillary exudates, B-scan ultrasonography revealed multiple vitreous opacities. We found no obvious abnormality in the left eye.

**Figure 1:**
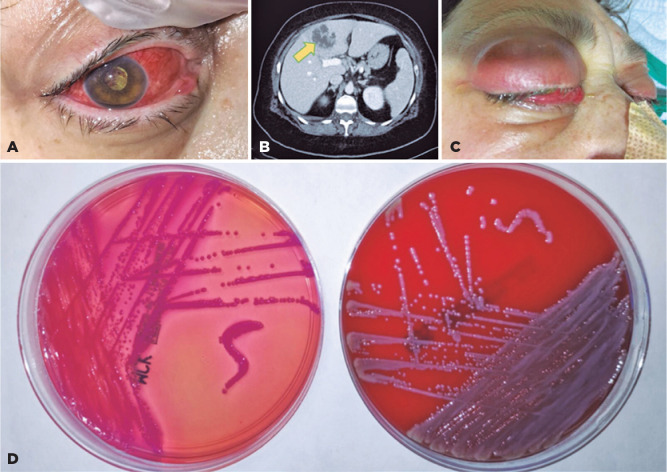
(A) Red eye and exudative plaque covering the pupil during the first examination. (B) Abdominal CT scan imaging showing a heterogeneous, hypodense, hypovascular liver lesion (yellow arrowhead), suggesting liver abscess. (C) Panophthalmic eye with upper eyelid edema and conjunctival chemosis during the examination in the critical care unit. (D) Vitreous cultures of suspected endophthalmitis: round and pink colonies on MacConkey agar plate (left) and round and gray colonies on blood agar plate (right) during the second day of incubation.

After making a provisional diagnosis of acute infectious endophthalmitis, we had the patient undergo aqueous and vitreous taps for culture, as well as intravitreal injection of antibiotics (10 mg/ml vancomycin and 20 mg/ml cefotaxime). We also had her started on 50 mg/ml fortified vancomycin and 100 mg/ml ceftazidime on an hourly basis. The blood count results revealed mild leukocytosis. Abdominal computerized tomography (CT) scan results showed a heterogeneous, hypodense, hypovascular liver lesion, suggesting liver abscess (Figure 1B). Because of this possibility, general surgeons performed ultrasound-guided percutaneous drainage of the liver lesion after empirical antibiotic therapy with ampicillin, sulbactam, and metronidazole. Abscess and blood cultures, as well as aqueous and vitreous fluids, revealed the presence of gram-negative bacilli (Figure 1D), later identified as *K. variicola* using matrix-assisted laser desorption/ionization-time of flight mass spectrometry (MALDI-TOF MS). *K. variicola* was susceptible to cefotaxime and ceftazidime but was resistant to ertapenem, meropenem, and imipenem. The patient was admitted to a critical care unit because of septic shock. Because of panophthalmitis with pus in the anterior chamber and a dense fibrin plaque over the pupil with no fundal details or visible red reflex (Figure 1C), we administered one more intravitreal injection of 10 mg/ml vancomycin and 20 mg/ml cefotaxime in the RE. The fibrin clot in her anterior chamber progressively resolved while the inflammatory material in the vitreous cavity became organized. However, at the end of a 12-month follow-up, the recorded BCVA was HM, and tonometry showed an IOP of 16mmHg with no signs of active intraocular inflammation.

Infections from *K. variicola*, an opportunistic gramnegative, facultative anaerobic, and nonmotile bacillus, and *Klebsiella pneumoniae* lead to bloodstream, respiratory tract, and urinary tract infections. In addition, *K. variicola* has been linked to infections in individuals with comorbidities, such as alcoholism, cancer, diabetes mellitus (as in this case), hepatobiliary diseases, and solid organ transplantation^(2)^. A previous study on 139 bloodstream infections reported that *K. variicola* infection had the highest mortality rate^(3)^. In addition, a recent study on an outbreak of neonatal sepsis found a significant mortality rate attributed to *K. variicola*^(4)^.

Endogenous endophthalmitis occurs when organisms infect the eye via the bloodstream and infiltrate the blood-ocular barrier into the internal ocular spaces. These cases are deemed medical emergencies as treatment delays may result in permanent loss of vision. In addition, outcomes are worsened by several factors: delay in diagnosis, use of inappropriate antibiotics, diffuse infection of the vitreous and retina or panophthalmitis, poor vision at presentation, and infection with virulent organisms or gram-negative bacteria^(5)^.

In conclusion, we report the first case of *K. variicola* infection that led to endogenous endophthalmitis. This species should be included in the list of microorganisms responsible for acute endogenous endophthalmitis. Clinicians must consider the overlap between ocular and systemic disease. Prompt diagnosis and treatment are essential to preserve vision; therefore, a high level of awareness of this disease is needed to prevent devastating consequences.

## References

[1] Durand ML. (2013). Endophthalmitis. Clin Microbiol Infect.

[2] Rodríguez-Medina N, Barrios-Camacho H, Duran-Bedolla J, Garza Ramos U. (2019). Klebsiella variicola: an emerging pathogen in
humans. Emerg Microbes Infect.

[3] Maatallah M, Vading M, Kabir MH, Bakhrouf A, Kalin M, Nauclér P (2014). Klebsiella variicola is a frequent cause of bloodstream infection
in the stockholm area, and associated with higher mortality compared to K.
pneumoniae. PLoS One.

[4] Farzana R, Jones LS, Rahman MA, Andrey DO, Sands K, Portal E (2019). Outbreak of hypervirulent multidrug-resistant klebsiella
variicola causing high mortality in neonates in Bangladesh. Clin Infect Dis.

[5] Jackson TL, Eykyn SJ, Graham EM, Stanford MR. (2003). Endogenous bacterial endophthalmitis: a 17-year prospective
series and review of 267 reported cases. Surv Ophthalmol.

